# Improvement of Fab expression by screening combinatorial synonymous signal sequence libraries

**DOI:** 10.1186/s12934-019-1210-1

**Published:** 2019-09-16

**Authors:** Antti Kulmala, Tuomas Huovinen, Urpo Lamminmäki

**Affiliations:** Department of Biochemistry/Biotechnology, University of Turku, Kiinamyllynkatu 10, 20520 Turku, Finland

**Keywords:** Codon usage, Fab fragment, Heterologous expression, *Escherichia coli*, PelB

## Abstract

**Background:**

Antibody fragments can be expressed in *Escherichia coli*, where they are commonly directed to the periplasm via *Sec* pathway to enable disulphide bridge formations and correct folding. In order to transport antibody fragments to the periplasmic space via *Sec* pathway, they are equipped with N-terminal signal sequence. Periplasmic expression has many benefits but it’s also subjected to many hurdles like inefficient translocation across the inner membrane and insufficient capacity of the translocation system. One solution to overcome these hurdles is a modulation of codon usage of signal sequence which has proved to be an efficient way of tuning the translocation process. Modulation of codon usage of signal sequences has been successfully employed also in improving the expression levels of antibody fragments, but unfortunately the effect of codon usage on the expression has not been thoroughly analyzed.

**Results:**

In the present study we established three synonymous PelB signal sequence libraries by modulating codon usage of light chain and heavy chain PelB signal sequences of a Fab fragment. Each region (n-region, hydrophobic region and c-region) of the PelB signal sequence in the both chains of the Fab fragment in a bicistronic expression vector was mutated separately. We then screened for clones with improved expression profile. The best source for improved clones was the n-region library but in general, improved clones were obtained from all of the three libraries. After screening, we analyzed the effects of codon usage and mRNA secondary structures of chosen clones on the expression levels of the Fab fragment. When it comes to codon usage based factors, it was discovered that especially codon usage of fifth leucine position of the light chain PelB affects the expression levels of Fab fragment. In addition, we observed that mRNA secondary structures in the translation initiation regions of the light and heavy chain have an effect on expression levels as well.

**Conclusions:**

In conclusion, the established synonymous signal sequence libraries are good sources for discovering Fab fragments with improved expression profile and obtaining new codon usage related information.

## Background

Antibody fragments, especially ScFv (single-chain variable fragment) and Fab (antigen binding fragment), are increasingly used for diagnostic and therapeutic purposes [1]. The antibody fragments can be expressed in *Escherichia coli*, where they are commonly directed to the periplasmic space by using *Sec* translocon to enable disulphide bridge formations and correct folding [2]. To this end, the expressed antibody polypeptides, which in the case of Fab consist of an intact light chain and the first two domains (V_H_ and C_H1_) of the heavy chain, are equipped with N-terminal leader (signal) sequences that guide them through the cytoplasmic membrane, most commonly, via the *Sec* translocon [3, 4].

Typically, the signal sequences are 25–30 residues long and they are generally composed of n-region, hydrophobic region and c-region [5]. The n-region has a positive charge and an average length of five (generally basic) residues, although the length highly varies. The hydrophobic region is 7–15 residues long and it adopts α-helical conformation. The c-region is composed of 3–7 neutral or polar amino acids, for example helix breaking proline and glycine residues and it also includes signal peptidase cleavage site. The c-region forms β-sheet structure [5, 6]. Many different signal sequences have been used to transport antibody fragments to periplasmic space of *E. coli* via *Sec* pathway [3], but also SRP dependent pathway has been utilized [7]. One of the most frequently used signal sequence for transportation of antibody fragments to the periplasm of *E. coli* is 22 amino acids long signal sequence of pectate lyase B (PelB) from *Erwinia carotovora* [8].

Compared to the cytosolic expression, the periplasmic expression required for antibody fragments (and proteins in general) is subjected to some hurdles like inefficient translocation across the inner membrane and insufficient capacity of the translocation system [9]. Various strategies have been described to increase periplasmic expression, one of which, involves the modulation of codon usage [10]. Zalucki et al. observed that non-optimal codons are required for expression and translocation of β-lactamase [11] and the same research group then showed that non-optimal codons in a signal sequence are necessary for the folding of the mature protein [12]. Controversially, it has been observed that non-optimal codons are enriched in the signal sequences of *Escherichia coli* on genomic level [13], but there is a report showing that optimal codons in some cases may be beneficial for improving the periplasmic expression especially with other secretory pathways than *Sec* [14]. Effects of the codon usage of signal sequences on the heterologous expression of antibody fragments have been previously studied by Stemmer et al. [15] who obtained increased expression of variable fragment (Fv) by introducing synonymous mutations in the second, heavy chain cistron signal sequence. In terms of translocation to the periplasm, Fab fragments are especially challenging since they are heterodimeric and both polypeptides are independently expressed and transported to the periplasm. Humphreys et al. showed that the optimization of the expression ratio of the light and heavy chain was important for the high level production of Fab fragment, and that the ratio can be modulated by synonymous mutations in the signal sequence [16]. In the study by Humphreys et al. synonymous mutations were introduced into the signal peptide of the major coat protein (VIII) of the phage M13 fused to the alkaline phosphatase gene and after identifying the best clones, the corresponding signal sequences were applied to Fab fragments. However, in the aforementioned studies, the effects of codon usage or mRNA secondary structure on the expression levels of given antibody fragment were not thoroughly analyzed by using the combination of codon usage metrics, mRNA folding algorithms and comprehensive statistical approaches.

Previously, we were able to increase expression of Fab fragment by harmonizing selected segments in the Fab fragment gene [17]. In the present study, we explore the effects of synonymous codon mutations introduced into the PelB signal sequences of light chain and heavy chain of a Fab fragment on protein expression in the periplasm. A separate combinatorial library of synonymous codons was created for the three structurally different regions of the signal peptide covering the N-terminal region (n-region), the hydrophobic core segment (hydrophobic region) and the hydrophilic C-terminal region (c-region), respectively. Unlike in the study conducted by Humphreys et al. we performed a combinatorial library screening directly with Fab fragments expressed from a bicistronic construct under *Lac* promoter. Clones with improved expression were found from all three sub-libraries and most notably in the n-region library. Particularly strong effect on expression was observed at the fifth position of the light chain signal sequence coding for a leucine, in which synonymous mutation caused almost 2-fold difference in expression. Moreover, the codon usage at fifth leucine position and mRNA secondary structures in the translation initiation region seemed to exhibit independent but additive roles on expression.

## Materials and methods

### Template sequences

**Fig. 1:**
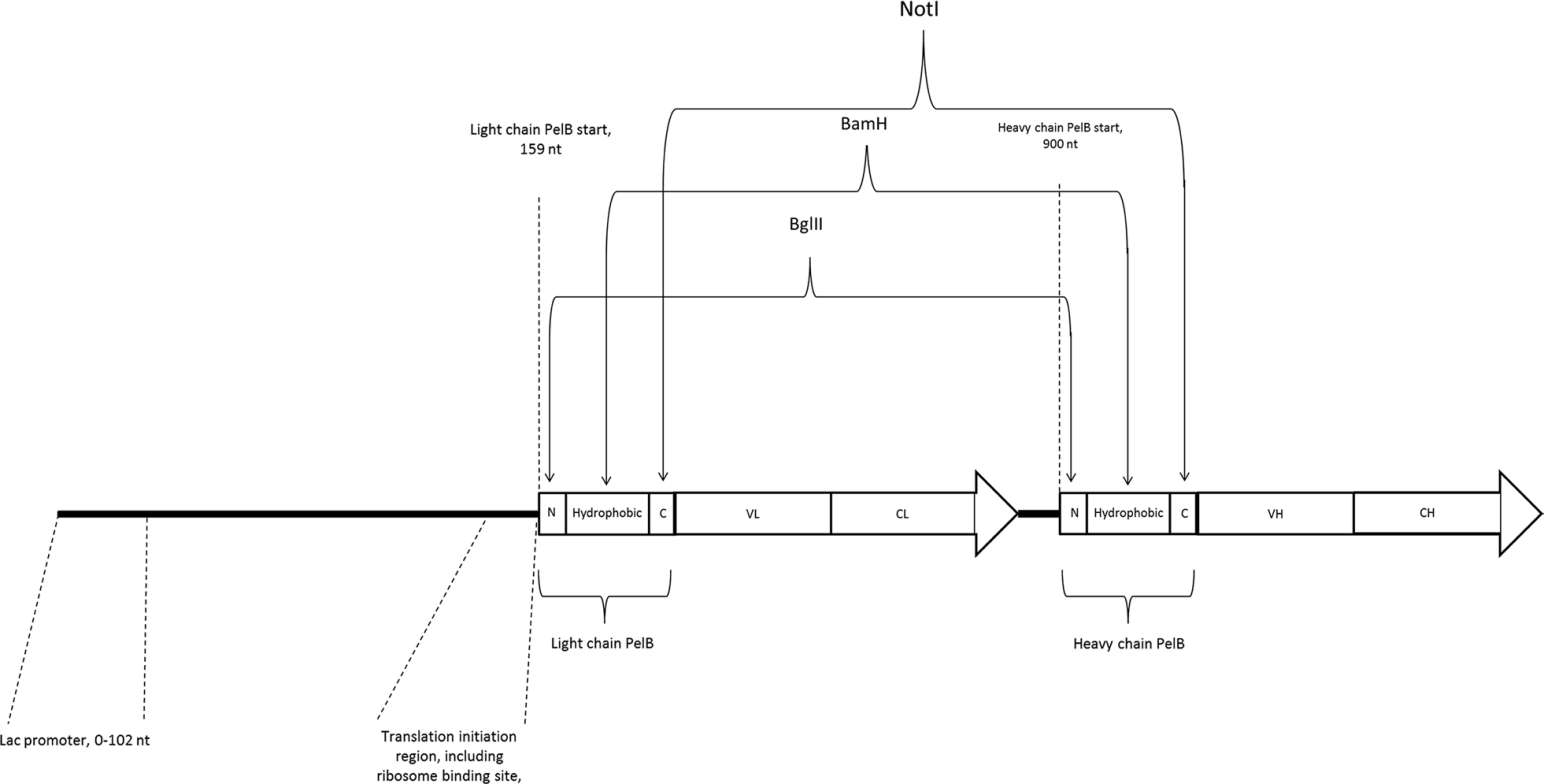
Schematic representation of bicistronic Fab template constructs. Braces with arrows point the recognition sites of the restriction enzymes BglII, BamHI and NotI present in the templates Fab0 N, Fab0H and Fab0C, respectively. Positions of *Lac* promoter, translation initiation region of the light chain, start position of the light chain PelB and the start position of the heavy chain PelB are indicated by dashed lines. *N* n-region, *Hydrophobic* hydrophobic region and *C* c-region

Fab0 N, Fab0H and Fab0C were used as templates in the construction of the libraries targeting the n- region, hydrophobic region and c-region of the signals sequences, respectively. These Fab constructs, which were based on the previously described anti-digoxigenin Fab0 fragment [17], carried BglII (Fab0N), BamHI (Fab0H) or NotI (Fab0C) restriction enzyme recognition site in PelB signal sequences of the light and heavy chains (Fig. 1). The restriction enzymes were used to abolish background sequences in the construction of the libraries. Hereafter, the libraries obtained by diversification of n- region, hydrophobic region and c-region are referred as N–0, H–0 and C–0, respectively.

### Production of uridylated single-stranded template DNA for Kunkel mutagenesis

For the production of phage stocks, Fab0 N, Fab0H and Fab0C in pEB32x vector [18] were transformed into *E. coli* XL1-Blue cells (Stratagene, LaJolla, USA) by electroporation as described by Huovinen et al. [19] and plated on LA plates (0.5% glucose, 25 µg/ml cm, 10 µg/ml tet). The plates were incubated overnight at 37 °C. Next day 3 × 5 ml of SB medium (1% glucose, 25 µg/ml cm, 10 µg/ml tet) was inoculated with one colony from appropriate plate and incubated overnight at 37 °C with 300 rpm shaking. Main culture of phage production and the phage precipitation were performed as described by Kulmala et al. [17]. Only exception was multiplicity of infection (MOI). In the production of pEB32x-Fab0N, pEB32x-Fab0H and pEB32x-Fab0C phage particles, the used MOI value was 10. Uridylated single-stranded template DNA was produced by using *E. coli* K12 CJ236 strain (NEB, Ipswich, USA) as described by Sidhu et al. [20]. Unlike in the protocol by Sidhu et al. the growth medium was supplemented with 6 µg/ml uridine.

### Kunkel mutagenesis for construction of N–0, H–0 and C–0 signal sequence libraries

Synonymous mutations were introduced to the n-region, hydrophobic region and c-region of the PelB signal sequences with phosphorylated primers that were purchased from Integrated DNA Technologies (Coralville, USA). Each region of the PelB signal sequences had its own designated primer (Additional file 1: Table S1). Phosphorylated primers were annealed to 700 ng of single-stranded pEB32x-Fab0N, pEB32x-Fab0H and pEB32x-Fab0C template sequences as described by Huovinen et al. [19]. Phosphorylated primers targeted to the light chain PelB and phosphorylated primers targeted to the heavy chain PelB were annealed in the same reaction. Remaining template sequences (background) in the Kunkel reactions pEB32x-N–0, pEB32x-H–0 and pEB32x-C–0 were digested in their entirety with 20 U BglII (Thermo Scientific), 20 U BamHI (Thermo Scientific) and 20 U NotI (Thermo Scientific), respectively. Buffer recommended by the manufacturer was used in each digestion reaction. All the digestion reactions were incubated for 2 h, at 37 °C and inactivated according to the manufacturer’s instructions. After inactivation, 2 U of uracil-DNA glycosylase (UDG) (Thermo Scientific) was added to each reaction. Reactions were incubated for 1 h, at 37 °C, purified with PCR purification kit and eluted into 20 µl.

### Selective rolling circle amplification (sRCA) and transformation of the N–0, H–0 and C–0 signal sequence libraries

All UDG treated Kunkel products were amplified in their entirety (20 µl) by selective rolling circle amplification (sRCA) in 50 µl total volume as described by Huovinen et al. [19] but with few exceptions. The amount of Phi29 Polymerase (Thermo Scientific) in the reactions was 25 U and in addition, 0.0625 U of inorganic pyrophosphatase (Thermo Scientific) was added to the reactions. The sRCA products were digested into linear single-plasmid segments with XhoI in 100 µl reactions containing 50 µl of DNA (sRCA reaction), 1× Buffer R (Thermo Scientific) and 100 U XhoI (Thermo Scientific). Reactions were incubated at 37 °C for 2 h, purified with PCR purification kit and eluted into 30 µl. After first XhoI digestion, additional XhoI digestions were performed by incubating overnight at 37 °C. Additional XhoI digestion reactions contained 30 µl of DNA, 1× Buffer R and 60 U XhoI in 60 µl volume. Digestion reactions were purified with PCR purification kit and eluted into 30 µl. Next, 30 µl of the digestion products were re-circularized in ligation reactions containing 5 ng/µl DNA, 0.02 U/µl T4 DNA ligase (Thermo Scientific) and 1× T4 DNA ligase buffer (Thermo Scientific). The ligation reactions were incubated overnight at 16 °C and 1 h at room temperature. The ligation products were purified with PCR purification kit, eluted into 25 µl and then transformed in their entirety into *E. coli* SS320 (MC1061 F′) cells [20] by electroporation. In three separate electroporations, 25 µl of DNA (~ 1.3 µg DNA in each ligation reaction) was mixed with 320 µl of cells and DNA was transformed to cells with Bio-Rad Genepulser (Bio-Rad, Hercules, USA) with settings 2.5 kV, 25 µF, 200 Ω. After transformation, cells were recovered in 25 ml of SOC medium for 1 h, at 37 °C, with 100 rpm shaking and subsequently plated on LA plates (0.5% glucose, 25 µg/ml cm, 10 µg/ml tet). Plates were incubated overnight at 37 °C. In addition, recovered cells were sampled and diluted 10^−1^–10^−6^ in 1 ml of SB medium for transformation efficiency calculations. Dilutions 10^−4^–10^−6^ were also plated on LA plates (0.5% glucose, 25 µg/ml cm, 10 µg/ml tet) and incubated overnight at 37 °C. The next day, cells were collected from the LA plates and stored as glycerol preps at − 70 °C.

### Cloning of the N–0, H–0 and C–0 signal sequence libraries from pEB32x vector into pEB07 vector

Signal sequence libraries N–0, H–0 and C–0 were cloned from pEB32x vector into pEB07 vector that fuses beta-lactamase gene (TEM-1) to the C-terminal end of a given molecule [19]. First, all three libraries were amplified in the PCR reactions (6 parallel reactions for each library) containing 1× Phusion HF reaction buffer (Thermo Scientific), 200 µM dNTP mix (Thermo Scientific), 1 U Phusion Hot Start II polymerase (Thermo Scientific), 10 ng of template DNA and 0.5 µM primers. Primer pair TH10 (5′-GATGGTAGAACGAAGCGG-3′) and TH40 (5′-CAGTAGTAGACGGCAGTGTCC-3′) was used. Thermal cycling condition was initial denaturation 98 °C for 1 min, denaturation 98 °C for 5 s, annealing 61.4 °C for 10 s, extension 72 °C for 31 s and final extension 72 °C for 5 min. PCR cycle was repeated 30 times. Reactions were pooled and the products purified with PCR purification kit and eluted into 30 µl. Next, the amplified libraries were digested with ApaI and XhoI restriction enzymes. All three libraries were digested in their entirety (30 µl) in 50 µl total volume containing 10 U ApaI (Thermo Scientific), 10 U XhoI (Thermo Scientific) and 1× CutSmart buffer (NEB). Vector pEB07 carrying Fab0 (pEB07-Fab0) gene was digested in the same manner. The digestion reactions were incubated overnight at 37 °C. Digested fragments were extracted from agarose gel with Gel Extraction kit (Thermo Scientific). Next, digested library fragments N–0, H–0 and C–0 were ligated to digested destination vector pEB07-Fab0 by using T4 DNA ligase (Thermo Scientific) according to manufacturer’s instructions. Molar ratio of inserts (library fragments) and vector (pEB07-Fab0) was 3:1. Ligation reactions were incubated overnight at room temperature and inactivated at 70 °C for 5 min. Then 20 µl (N–0), 25 µl (H–0) and 30 µl (C–0) of DNA were mixed with 280 µl of *E. coli* SS320 (MC1061 F’) cells in three separate electroporations with the same settings as described above. After transformation, cells were recovered in 20 ml of SOC medium, which had lowered glucose concentration (0.05%), for 1 h at 37 °C with 100 rpm shaking and subsequently plated on LA plates (0.05% glucose, 100 µg/ml amp). Plates were incubated overnight at 37 °C. Sampling and dilution of recovered cells was done in the same manner as described earlier. The next day, cells were collected from the LA plates and stored as glycerol preps at − 70 °C.

### Cultivation of the libraries in pEB07 vector in order to reduce the amount of non-productive clones

All three libraries were cultured in pEB07 vector in order to reduce the amount of non-productive clones in the libraries. In pEB07 vector, beta-lactamase gene is fused to the C-terminal end of the heavy chain of the Fab and thus expression of the heavy chain is linked to the fitness of the cell. Therefore, for example frameshifts resulting in a premature stop codon in the heavy chain will lead to cell death due to the loss of ampicillin resistance. Cultures were started by diluting glycerol preps to OD (600 nm) 0.1 in 5 ml of SB medium (25 µg/ml cm, 100 µg/ml amp). Cells were cultured at 37 °C with 300 rpm shaking until OD (600 nm) reached 0.5. Then, 2.5 ml of cells were taken and cells were pelleted by centrifugation (3220*g*, 3 min, at room temperature). After centrifugation, cells were re-suspended in 5 ml of fresh SB medium into OD (600 nm) 0.25. Culturing was continued at 37 °C with 300 rpm until OD (600 nm) reached 0.5 and subsequently the same dilution procedure was repeated. Every time when cells were re-suspended in fresh SB medium the concentration of ampicillin increased step-by-step. Cells were cultured for 10 generations in ampicillin concentrations 100, 200, 400, 600, 800, 1000, 1200, 1500, 2000 and 2500 µg/ml. After each generation, 800 µl samples were taken and samples were stored as glycerol preps at − 70 °C.

### Primary screening of the libraries on 96-well plates

Single colony primary screenings were performed on 96-well plates by expressing the clones of the libraries from pLK04 vector [21] that allows production of Fabs in soluble form with mere 6xhistidine tag fused to the C-terminus of heavy chain. All three libraries N–0, H–0, C–0 and pEB07-Fab0 were amplified by PCR in the reactions containing 1× Phusion HF reaction buffer (Thermo Scientific), 200 µM dNTP mix (Thermo Scientific), 1 U Phusion Hot Start II polymerase (Thermo Scientific), 1 ng of template DNA and 0.5 µM primers (TH10 and TH40). Cycling conditions were the same as mentioned above. Reactions were purified with PCR purification kit and eluted into 20 µl. After purification all four reactions were digested with ApaI and XhoI restriction enzymes as mentioned above. Also pLK04-Fab0, which was used as a destination vector, was digested in the same manner. Fragments were extracted from the gel with the gel extraction kit. The fragments from the libraries N–0, H–0, C–0 and pEB07-Fab0 were ligated to digested pLK04-Fab0 destination vector in the same manner as above. The ligation reactions were then transformed into CaCl_2_ competent *E. coli* XL1-Blue cells by heat shock. First, 10 µl of DNA was mixed with 100 µl of cells in each reaction and subsequently DNA–cell mixtures were put on ice for 30 min. After incubation on ice the heat shock was given to each transformation reaction at 42 °C for 90 s and subsequently 900 µl of SOC medium was added to each reaction. Cells were then recovered at 37 °C with 100 rpm shaking for 45 min. Cells were plated on LA plates (0.5% glucose, 100 µg/ml amp) and incubated overnight at 37 °C. The next day, colonies were picked from the plates to the wells of 96-well plates (Sarstedt, Nümbrecht, Germany). From each plated library, 279 colonies were picked (3 plates per library, 837 colonies in total) and colonies were inoculated to 160 µl of SB medium (1% glucose, 100 µg/ml amp). Twelve wells from each plate were reserved for Fab0 that was used as a parental expression control. Plates were covered with breathable tape (Nunc, Roskilde, Denmark) and incubated overnight at 37 °C with 900 rpm shaking with 70% humidity. After overnight incubation, cultures were diluted by pipetting 4 µl from the overnight cultures to 200 µl of fresh SB medium (0.05% glucose, 100 µg/ml amp). Refreshed cultures were incubated for 4 h at 37 °C with 900 rpm shaking with 70% humidity. After 4 h of incubation, 10 µl of 4 mM IPTG was added to the wells and cultures were continued overnight at 26 °C with 900 rpm shaking. The next day 10 µl samples, which were used for sequencing, were taken from each well. After sampling, 20 µl of 10× Lysis buffer (1× TBS [50 mM Tris–HCl, pH 7.5; 150 mM NaCl], 10 mg/ml lysozyme from chicken egg white [Alfa Aesar, Haverhill, USA], 25 U/ml universal nuclease for cell lysis [Thermo Scientific]) was added to the wells. Plates were incubated at room temperature with slow shaking for 30 min and subsequently subjected to 1 freeze–thaw cycle. The expression levels of immunoreactive Fab were measured with time-resolved fluorometry based immunoassay as described below. Immunoassay was implemented mainly the same way as in the study by Kulmala et al. [17].

In the primary screening, the wells of streptavidin plates (Kaivogen, Turku, Finland) were coated with 100 µl per well of 500 nM biotinylated digoxigenin. The streptavidin plates were incubated at RT for 1 h with slow shaking and subsequently washed two times with Delfia Plate Wash (Wallac, Turku, Finland) by using Kaivogen wash buffer (Kaivogen). After washing, 10 µl of samples were diluted in 100 µl of Assay Buffer (Kaivogen). Samples were diluted directly in the wells of streptavidin plate. After the addition of samples, the plates were incubated at RT for 1 h with slow shaking and subsequently washed two times. Immunoreactive Fabs were detected with 25 ng per 100 μl N1-Eu-chelate labelled anti-human Fab 2A11 (Hytest Ltd, Turku, Finland) and by incubating at RT for 1 h with slow shaking. The streptavidin plate was washed four times. Delfia Enhancement solution (Wallac) was added and the plate was incubated at RT for 10 min with slow shaking, after which time-resolved europium signal at 615 nm was measured with Victor 1420 Multilabel Counter (Wallac).

### Secondary screening of the libraries

The clones were ranked based on the signal levels obtained from primary screening. Selected clones from the top 10% cohort were subjected to secondary screening. These clones consisted of 30 N–0 library clones, 10 H–0 library clones and 18 C–0 library clones. The secondary screening cultures were started by diluting overnight pre-cultures to OD (600 nm) 0.1 in 5 ml of SB medium (1% glucose, 100 µg/ml amp). Cells were cultured at 37 °C with 300 rpm shaking to OD (600 nm) 0.7–0.9. When the cell cultures reached the appropriate OD (600 nm), the same amount of cells (2 × 10^9^ cells) were taken from each culture and subsequently cells were centrifuged at 3220*g* for 11 min at 20 °C. After centrifugation the supernatant was discarded and pellets dissolved in 5 ml of fresh SB medium (1 mM IPTG, 100 µg/ml amp) to OD (600 nm) 0.5. Cells were cultured at 26 °C with 300 rpm shaking for 3 h and subsequently 1 ml sample was taken from each culture. Cells were pelleted with tabletop centrifuge at 16,300*g*, for 5 min at 4 °C. Cell pellets were suspended in Assay Buffer and cells were disrupted by sonication while keeping on ice.

The yields of immunoreactive Fab fragments were measured by immunoassay. The procedure of the immunoassay has been previously described by Kulmala et al. [17]. Wells of a 96 well streptavidin plate were coated with 100 μl per well of 1 μM biotinylated digoxigenin in Assay Buffer. The streptavidin plate was incubated at RT for 1 h with slow shaking. After incubation, the streptavidin plate was washed two times. After washing, 100 μl per well of samples were added as triplicate. Samples were first diluted 1/100 in Assay Buffer before addition on the wells. For the quantification of immunoreactive Fab, 100 μl per well of purified Fab0 was used as a standard at final concentrations of 0; 0.0015; 0.003; 0.015; 0.03; 0.15; 0.3 μg/ml in Assay buffer. The streptavidin plate was incubated at RT for 1 h with slow shaking and subsequently washed two times. Immunoreactive Fabs were detected with 25 ng per 100 μl N1-Eu-chelate labelled anti-human Fab 2A11 and by incubating at RT for 1 h with slow shaking. The streptavidin plate was washed four times. Delfia Enhancement solution was added and the plate was incubated at RT for 10 min with slow shaking, after which time-resolved europium signal at 615 nm was measured with Victor 1420 Multilabel Counter.

### Up-scaled Fab fragment expression

The anti-digoxigenin Fab fragment equipped with the best library clone PelB (N–0 2) (Additional file 1: Fig S1) and the parental PelB were expressed in 500 ml culture volume in three replicates in the same manner as the secondary screening of the libraries. The only exception was the OD (600 nm) of the induction which in this case was 0.6. After induction, 1 ml sample was taken from each culture and cells were pelleted as described above. The pellets were re-suspended in periplasmic extraction buffer and periplasmic extraction was implemented as described by Humphreys et al. [16]. Subsequently, the cells were pelleted and periplasmic extracts were collected. The pelleted cells were re-suspended in Assay Buffer and sonicated.

### Colony PCR for sequencing

The top ranking 10% cohort of clones (see “secondary screening of the libraries”) was sequenced and for these purposes, Fab DNA from the cell samples taken after primary screening was amplified by colony PCR. First, the cell samples were diluted in 1/16 in MQ water. Then the samples were amplified in the reactions containing 1× Taq buffer (NH_4_)SO_4_ (Thermo Scientific), 200 µM dNTP mix (Thermo Scientific), 0.5 U Taq DNA polymerase (Thermo Scientific), 0.5 µl of diluted cell sample and 0.5 µM primers. Primer pair TH34 (5′-AAGGGCAATCAGCTGTTG-3′) and pAKrev (5′-CGCCATTTTTCACTTCACAG-3′) was used. Thermal cycling condition was initial denaturation 95 °C for 3 min, denaturation 95 °C for 30 s, annealing 48.5 °C for 30 s, extension 72 °C for 1 min and final extension 72 °C for 10 min. PCR cycle was repeated 34 times. The reactions were purified in enzymatic purification reactions containing 4 µl of PCR product, 0.8 µl of FastAP (1 U/µl) (Thermo Scientific), 0.4 µl of Exonuclease 1 (20 U/µl) (Thermo Scientific). Reactions were incubated at 37 °C for 15 min and inactivated at 85 °C for 15 min. After purification the samples were sequenced by Macrogen Inc. (Seoul, South Korea).

### Sequence analysis

Sequence analysis was implemented by using programmable stand-alone software called Visual Gene Developer [22]. Visual Gene Developer enables analysis of different sequence variables like codon usage and mRNA secondary structures. Local Gibbs free energy (strength of secondary structure) of mRNA was calculated by using “mRNA profile v2” module that uses sliding window analysis in order to determine local Gibbs free energy of mRNA in different parts of a test sequence. Selected window size was 30 and step size was 1. For the codon usage analysis we used two codon usage metrics, relative adaptiveness values (*w*_*i*_) and CAI (codon adaptation index). The relative adaptiveness value of a codon is the usage frequency of the codon compared to the most frequently used codon encoding the same amino acid. The most frequently used codon encoding a given amino acid has the relative adaptiveness value of 1 [23]. The relative adaptiveness values were obtained from *Escherichia coli* K-12 codon table compiled by Visual Gene Developer. The Visual Gene Developer retrieves the codon usage frequency data from CUTG (Codon Usage Tabulated from GenBank) (www.kazusa.or.jp/codon/) and calculates relative adaptiveness values for each codon by using the codon usage frequency data obtained from CUTG. The CAI value is simply a geometrical mean of individual relative adaptiveness values [23]. The relative adaptiveness values were used to assess the effect of individual codon positions on the expression levels of Fab fragment, whereas the CAI values were utilized when the effect of codon pairs and the codon usage of whole regions on expression levels were studied.

### Statistical analysis

All statistical analyses were performed with IBM SPSS Statistics 22 (Armonk, USA). All statistical analyses that were used for the interpretation of results are shown and explained simultaneously with the representation of the results.

## Results

### Design of the synonymous PelB signal sequence libraries

**Fig. 2:**
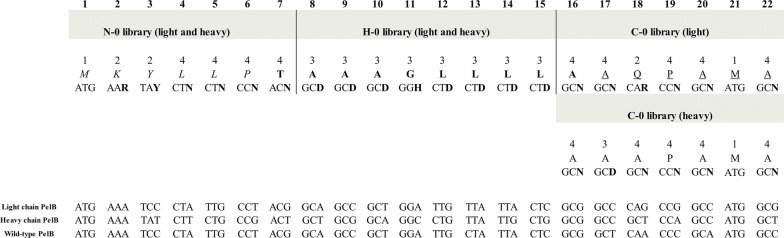
The design of the signal sequence libraries. The boundaries of each library are marked with vertical bars. The amino acid sequence is shown above the codon sequence. Numbers above the amino acid sequence illustrate the positional diversity. Bold numbers above the table show the position number for each amino acid in the sequence. Letters in italics denote the natural n-region, bold letters denote the natural hydrophobic region and underlined letters denote the natural c-region. The parental light chain PelB, the parental heavy chain PelB and the wild-type PelB codon sequences are shown. **H** = C, T, A; **D** = A, G, T; **Y** = C, T; **V** = A, G, C; **R** = A, G

As a model system we used a codon-harmonized anti-digoxigenin Fab fragment, expressed in a bicistronic vectors under *Lac P/O* with both the light and heavy chain preceded by a PelB signal sequence. Codon sequences of parental light chain PelB, parental heavy chain PelB and wild-type PelB (GenBank Accession number: S51475) are shown in Fig. 2. Three synonymous signal sequence libraries, N–0, H–0 and C–0, were designed and established by mutating the third base of a codon in either the n-region, hydrophobic region or c-region of the PelB signal sequences, respectively (Fig. 2). The given region was simultaneously diversified in both the light and the heavy chain PelB of the Fab. To facilitate the construction of the libraries and covering of theoretical diversity, few restrictions in the library designs were made. The restrictions included, first of all, exclusion of TTA and TTG codons from leucine codon repertoire. Diversity was restricted also in the hydrophobic region, where variation at the third base position of each codon was limited to 3 bases (Fig. 2). In addition, the first threonine and the last alanine positions of the hydrophobic region were relocated in the N–0 library and C–0 library, respectively (Fig. 2). Diversity of the third base in the codon for the second alanine position in the c-region of the heavy chain PelB was also limited to 3 bases. In the situations where positional diversity was restricted to three codons, the criterion for the exclusion was the similarity of codon usage frequencies between the codons. If two codons had codon usage frequencies close to each other, one of them was excluded. The restrictions were based on the codon usage frequency table of *Escherichia coli* K12 on Kazusa codon usage database [24]. The theoretical diversities of the N–0, H–0 and C–0 libraries were 1.05 × 10^6^, 4.3 × 10^7^ and 6.3 × 10^6^, respectively. Sizes of the N–0, H–0 and C–0 libraries in pEB32x vector were 4.52 × 10^8^ cfu, 4.86 × 10^8^ cfu and 4.50 × 10^7^ cfu, respectively. After libraries were cloned from pEB32x vector into pEB07 vector the library sizes were 7.56 × 10^7^ cfu, 8.40 × 10^7^ cfu and 3.68 × 10^7^ cfu, respectively.

### The highest expressing variants were obtained from the N–0 library

**Fig. 3:**
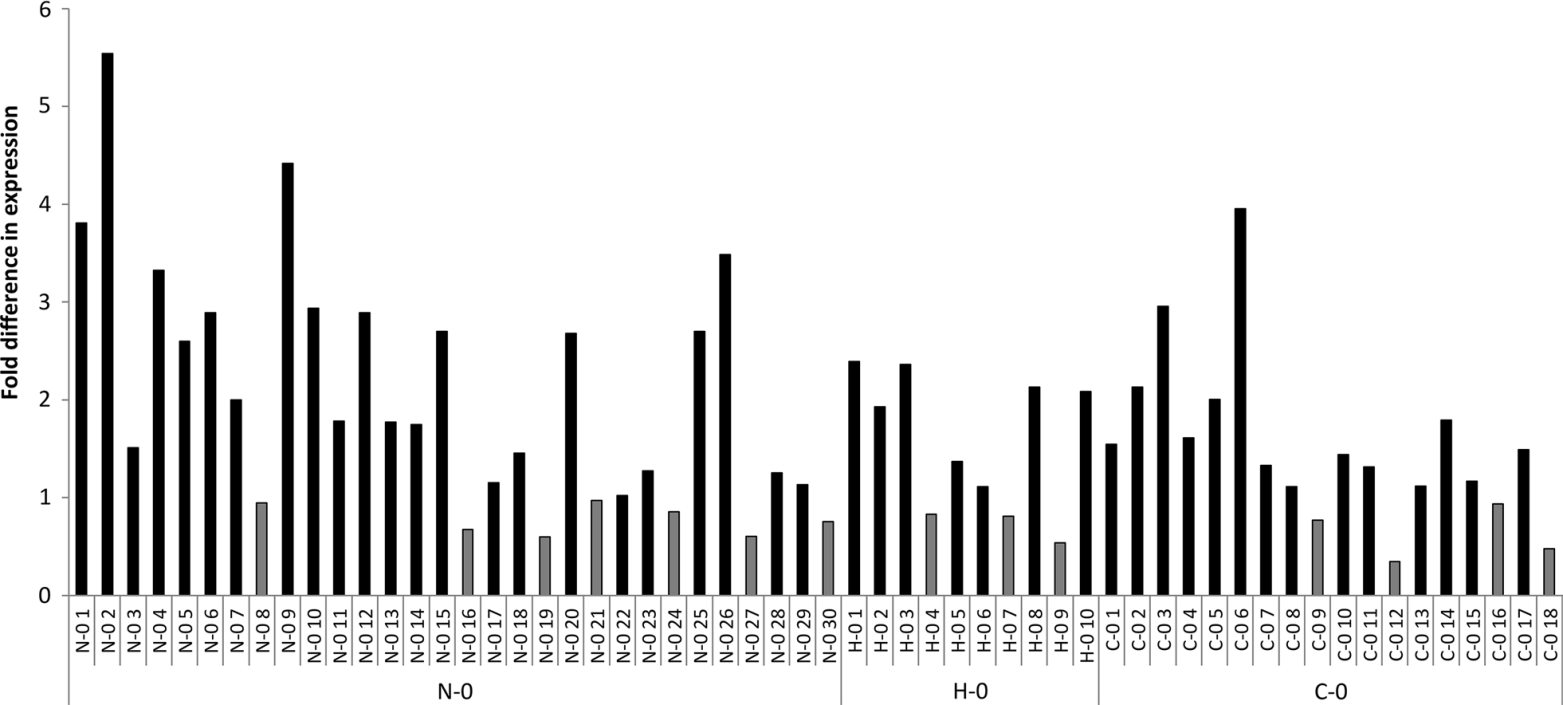
Fold difference in expression compared to the parent Fab0. The libraries and clones of each library are shown on the x-axis and separated by vertical lines. Gray bars indicate decreased expression compared to the parent Fab0

The libraries were initially expressed as a fusion with beta-lactamase and subjected to antibiotic selection to enrich functional clones. The enriched Fab genes were cloned to another bacterial expression vector to produce soluble Fab fragments for the primary screening for expressions levels. Altogether, 837 individual clones were screened with time-resolved fluorometry based immunoassay for the expression level of immunoreactive Fab and sorted according to the obtained signal levels. Top 10% cohort consisted of 40 N–0 library clones, 21 H–0 library clones and 23 C–0 library clones. All the clones belonging to the top 10% cohort and the parental Fab0 constructs included as controls in the screening cultures were sequenced. The library clones carrying valid signal sequences (no insertions, deletions, transitions or transversions) were directed to secondary screening. All clones having background (i.e. template) sequences in both the chains were also ignored from secondary screening. However, if only one chain contained background sequence, the variant was included into the set. The remaining clones consisted of 30, 10 and 18 Fab constructs from the N–0, H–0 and C–0 libraries, respectively. Codon sequences of the clones are shown in Additional file 1: Fig. S1. To this end, the cells were cultured in 5 ml volume that enabled tighter control over the variables affecting expression than the primary screening cultures performed on 96-well plates. Based on the secondary screening results, the best performing library in terms of increased expression was N–0 where 43% of the clones exhibited at least twofold increase and 17% at least threefold increase in expression compared to the parent Fab0 (Fig. 3). The corresponding values for the H–0 library were 40% and 0%, respectively and for C–0 library 22% and 6%, respectively. In the libraries N–0, H–0 and C–0, 23%, 30% and 22% of the clones showed decreased expression, respectively. The highest expressing variant, which exhibited over fivefold higher expression levels than the parent Fab0, was obtained from the N–0 library. The average expression of the parent Fab0 was 0.56 µg/ml (n = 6).

### Codon optimality of Leu-5 of light chain PelB affects the amount of functional Fab fragment

**Fig. 4:**
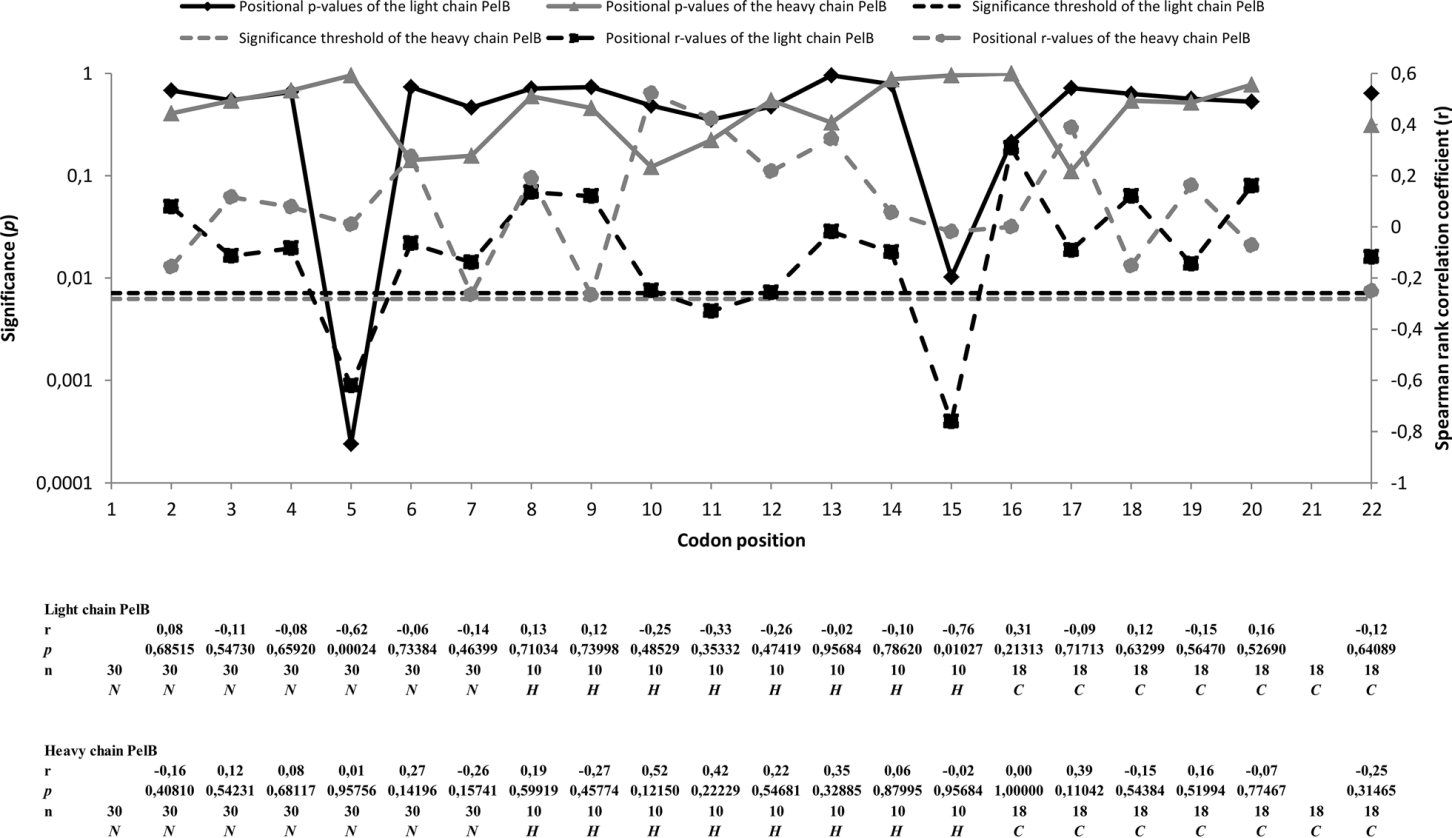
Correlation between the relative adaptiveness values and the expression levels of immunoreactive Fab fragment. Black line (with diamond markers) indicates the *p*-value at given position in the light chain PelB and grey line (with triangle markers) indicates the *p*-value at given position in the heavy chain PelB. Dashed black line (with square markers) indicates the Spearman rank correlation coefficient at given position in the light chain PelB and dashed grey line (with sphere markers) indicates the Spearman rank correlation coefficient at given position in the heavy chain PelB. Horizontal dashed black and grey lines indicate the levels below which the correlations are significant after Bonferroni correction for multiple correlations in N–0 library (0.00714) and in H–0 library (0.00625), respectively. Positions where solid black or grey line goes below the dashed vertical lines are considered as statistically significant. Spearman rank correlation coefficients (r), *p*-values and sampling sizes (n) of each correlation analysis at each position are shown in numerical form below the graph. Letters *N*, *H* and *C* denote the N–0, H–0 and C–0 library regions, respectively

To closer investigate the relation of the codon usage and the expression of the Fab, we analyzed the codon usage of the signal sequence libraries by using the relative adaptiveness values (*w*_i_) of each codon and the CAI value which is a geometrical mean of relative adaptiveness values [23]. Relative adaptiveness value of a codon is the usage frequency of the codon compared to the most frequently used codon encoding the same amino acid. The most frequently used codon encoding a given amino acid has the relative adaptiveness value of 1. Traditionally, the most frequently used codons are considered to be the most optimal ones [25]. Relative adaptiveness values for each codon were obtained from the *Escherichia coli* K-12 codon usage table compiled by Visual Gene Developer. The relative adaptiveness values were used when the effect of individual codon positions on the expression levels was assessed. The CAI value was used when the effect of codon pairs or the codon usage of whole regions on the expression levels was analyzed. We calculated Spearman rank correlation coefficient between the relative adaptiveness values and the expression levels of immunoreactive Fab fragment at each codon position in each library (Fig. 4). The analysis was implemented by plotting the expression levels of the clones in the libraries N–0 (n = 30), H–0 (n = 10) and C–0 (n = 10) against the relative adaptiveness values at each codon position, resulting in the same number of correlation coefficients as there were codon positions in the library. After Bonferroni correction for multiple correlations, the analysis revealed that leucine position Leu-5 of the light chain PelB in the N–0 library (PelB n-region: MKYLLP) correlated significantly with the expression (n = 30, r = − 0.622, 2-tailed *p *= 0.00024) (Fig. 4). In addition, significant negative correlation was observed at leucine position Leu-15 of the light chain PelB in the H–0 library (PelB hydrophobic region: TAAAGLLLLA) (n = 10, r = − 0.763, 2-tailed *p *= 0.01), but the significance was lost after the Bonferroni correction (Bonferroni corrected alpha level= 0.00625) (Fig. 4). No correlations between relative adaptiveness values and expression levels of immunoreactive Fab fragment were found in C–0 library. Moreover, significant correlations were not found in the heavy chain PelB signal sequence.

**Fig. 5:**
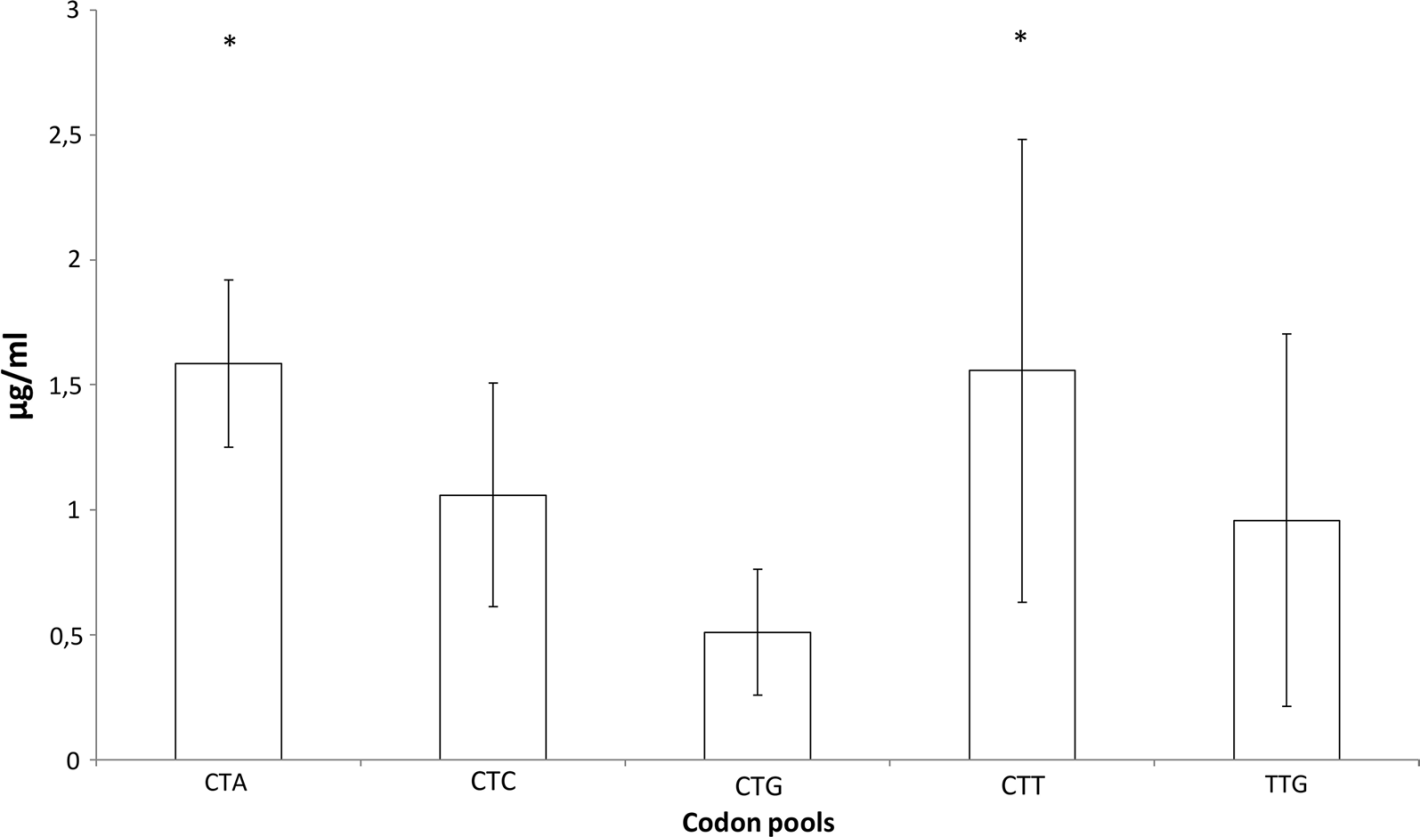
Effect of leucine codons on expression at position Leu-5 in N–0 library. The clones in N–0 library were pooled according to the codon identity at position L5 (CTA, CTC, CTG, CTT or TTG). The average expression levels and the standard deviation of each pool are represented. According to the Kruskal–Wallis analysis, CTG codon (n = 6) at position Leu-5 caused significant decrease in expression when compared to CTA codon (n = 6) (Adj. Sig. *p* = 0.005) and CTT codon (n = 7) (Adj. Sig. *p* = 0.035) in N–0 library. Sampling sizes of CTC and TTG codons were 7 and 5, respectively. Statistically significant (*p *< 0.05) differences to CTG codon are indicated with asterisk (*)

Next, we pooled the clones in N–0 library according to the codon identity at position Leu-5 (CTA, CTC, CTG, CTT or TTG) and we calculated average expression levels for each group. Closer look at position Leu-5 revealed that the most optimal leucine codon CTG (*w*_*i*_ = 1) is especially deleterious for the expression of immunoreactive Fab fragment, and furthermore, Kruskal–Wallis analysis showed that the less optimal leucine codons CTA (*w*_*i*_ = 0.0724) and CTT (*w*_*i*_ = 0.2093) have significantly higher expression levels of immunoreactive Fab fragment than CTG codon (Fig. 5).

In addition to addressing the optimality of individual codons, we analyzed that of codon pairs (CAI of the codon pair) in each library and plotted the obtained values against the expression levels of immunoreactive Fab fragment. Codon pairs that correlated with the expression levels were observed only if one of the codons in the codon pair was at the position Leu-5. According to the Spearman rank correlation coefficient, both correlating codon pair positions, [Leu-4]-[Leu-5] (n = 30, r= − 0.511, *p* = 0.004) and [Leu-5]-[Pro-6] (n = 30, r= − 0.486, *p* = 0.006), showed negative correlation with the expression. As observed with single codons, non-optimal codon also in codon pairs increased the expression levels. Bonferroni corrected *p*-values for both correlations were 0.0167 and 0.0125, respectively.

After analyzing the positional correlations between the relative adaptiveness values and the expression levels of immunoreactive Fab fragment, we calculated CAI values for the light and heavy chain PelB signal sequences of each clone in each library. Then the CAI values of the light and heavy chain PelB signal sequences were plotted against expression levels of immunoreactive Fab fragment by using Spearman rank correlation coefficient. In the case of N–0 library, no correlations were observed either in the light (n = 30, r = − 0.354, 2-tailed *p *= 0.055) or in the heavy chain PelB (n = 30, r = − 0.040, 2-tailed *p *= 0.834). For the H–0 library, correlation between the CAI and expression level of immunoreactive Fab fragment was significant in the light chain PelB (n = 10, r = − 0.697, 2-tailed *p *= 0.025). Non-optimal codons increased expression levels in this case as well. However, the correlation was lost (n = 10, r = − 0.360, 2-tailed *p *= 0.307) when the position Leu-15 was not taken into account. Furthermore, in the H–0 library, there was no correlation between the CAI and expression level of immunoreactive Fab fragment in the heavy chain PelB (n = 10, r = 0.250, 2-tailed *p *= 0.486). In the C–0 library, significant correlation between the CAI and expression level of immunoreactive Fab fragment was not observed in the light chain PelB (n = 18, r = − 0.059, 2-tailed *p *= 0.816) or in the heavy chain PelB (n = 18, r = − 0.035, 2-tailed *p *= 0.890).

**Fig. 6:**
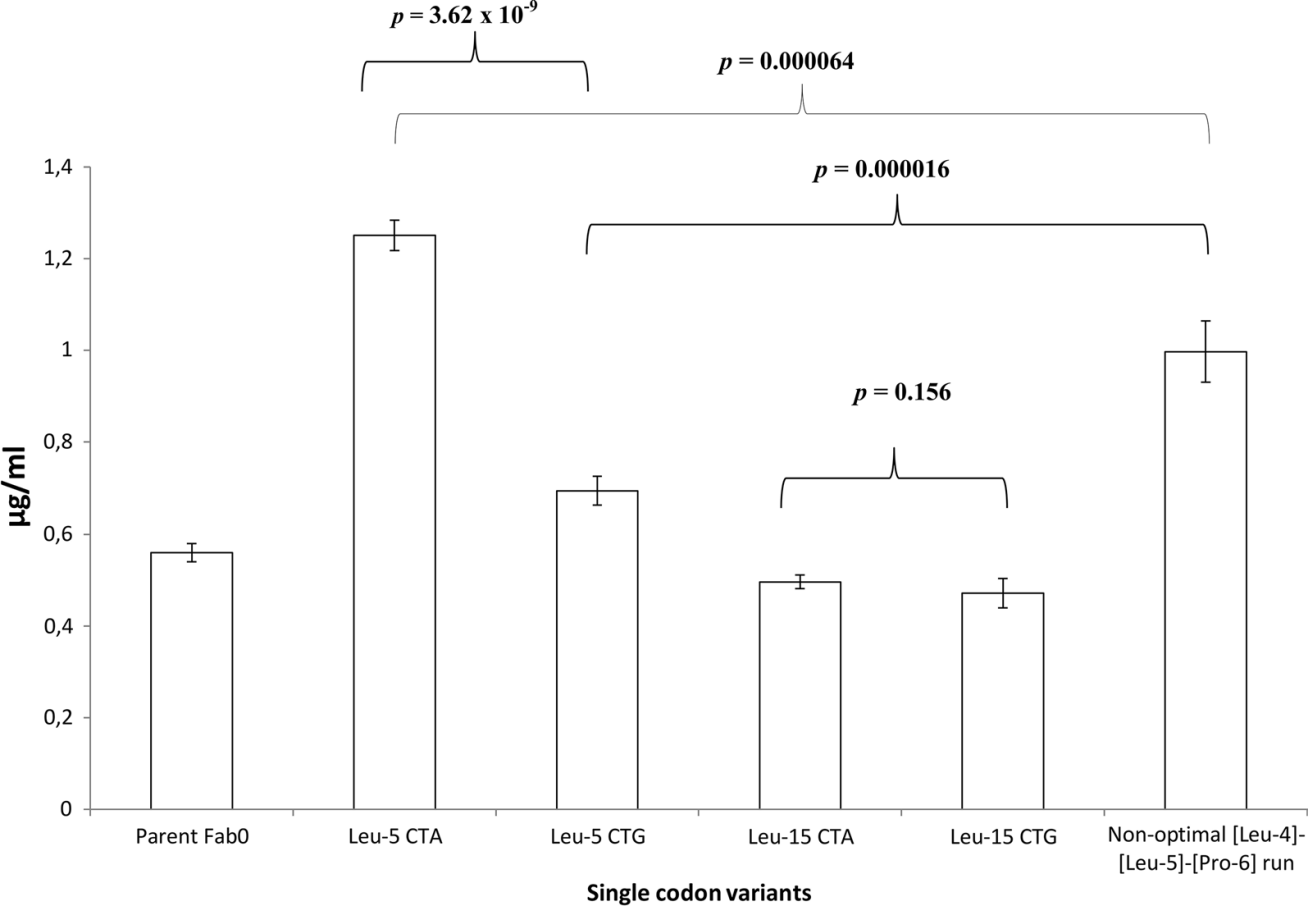
Average expression levels of single codon variants. Each expressed single codon variant and parent Fab0 are represented on the x-axis. The average expression levels and the standard deviation from five independent expressions are represented. The average expression levels of single codon variant Leu-5 CTA was compared to Leu-5 CTG and non-optimal [Leu-4]-[Leu-5]-[Pro-6] codon run variant. The average expression levels of single codon variant L15 CTA was compared to Leu-15 CTG. In addition Non-optimal [Leu-4]-[Leu-5]-[Pro-6] codon run variant was compared to the Leu-5 CTG variant. All comparison combinations are indicated by braces and *p*-values of the comparisons are shown above the braces

To confirm the findings made at the position Leu-5, we generated single codon variants by introducing CTA (the least optimal) or CTG (the most optimal) codon to the Leu-5 position of the light chain PelB of the parent Fab0 and compared the expression levels. Same mutations were introduced also to the position Leu-15. After five independent parallel expressions, significant (*T* test, Equal variances assumed, Sig. 2-tailed *p* = 3.62 × 10^−9^) difference was observed between Leu-5 CTA variant and Leu-5 CTG variant (Fig. 6). The Leu-5 CTA variant exhibited on average 1.8-fold higher expression than the Leu-5 CTG variant (Fig. 6). However, the difference between the variants Leu-15 CTA and Leu-15 CTG was insignificant (T-test, Equal variances assumed, Sig. 2-tailed *p* = 0.156) (Fig. 6). Since it was observed that non-optimal [Leu-4]-[Leu-5] and [Leu5]-[Pro-6] codon pairs increased expression levels, we also produced a variant containing non-optimal codon run CTA–CTA–CCC (CCC codon is the least optimal proline codon) at position [Leu-4]-[Leu-5]-[Pro-6] and compared its average expression levels to that of the Leu-5 CTA variant. Surprisingly, the non-optimal codon run variant exhibited 1.3-fold lower average expressions than the Leu-5 CTA variant (Fig. 6). The difference was also statistically significant (T-test, Equal variances assumed, Sig. 2-tailed *p* = 0.000064). Although the non-optimal codon run variant exhibited lower average expression than the Leu-5 CTA variant, it however, exhibited significantly (T-test, Equal variances assumed, Sig. 2-tailed *p* = 0.000016) higher average expression (1.4-fold) than the Leu-5 CTG variant (Fig. 6).

Finally we compared the average expression levels of the single codon variants Leu-5 CTA and Leu-5 CTG to the average expression levels of Leu-5 CTA and Leu-5 CTG codon pools. According to T-test, there were no significant differences in expression levels between the single codon variant Leu-5 CTA and Leu-5 CTA codon pool (T-test, Equal variances assumed, *p* = 0.054) or the single codon variant Leu-5 CTG and Leu-5 CTG codon pool (T-test, Equal variances not assumed, *p* = 0.134). The results show that the expression data regarding position Leu-5 in N–0 library is valid also when only codons at position Leu-5 are altered, further highlighting the importance of the position Leu-5.

### Secondary structures of the mRNA in translation start sites of the light chain and the heavy chain affect the expression levels of immunoreactive Fab fragment, but in different ways

**Fig. 7:**
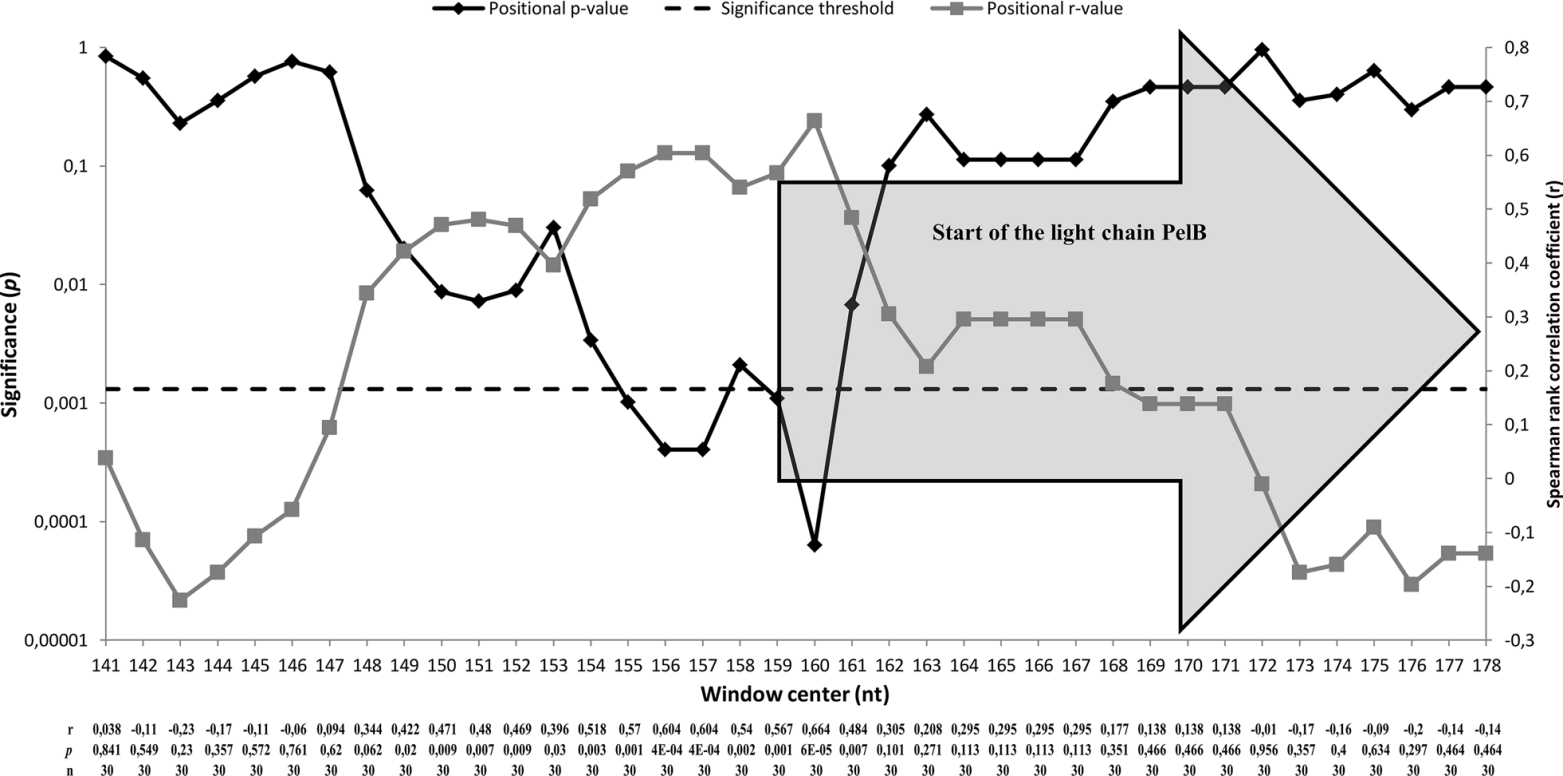
Correlation between positional Gibbs free energy of mRNA secondary structure and Fab expression in the light chain region of the N-0 library. Black line indicates the *p*-value at given position and dashed black line indicates the level below which the correlation is significant. Grey line indicates the Spearman rank correlation coefficient value at given position. The grey arrow denotes the start and the translational direction of the light chain PelB. Spearman rank correlation coefficients (r), *p*-values and sampling sizes (n) of each correlation analysis at each position are shown in numerical form below the graph

In many cases it has been shown that mRNA secondary structures near the translation start site play a major role when it comes to the expression of proteins. For example, Osterman et al. showed that stable secondary structures sequestering the Shine-Dalgarno (SD) sequence and start codon inhibit expression [26]. In addition, Kudla et al. determined that mRNA folding energy in a region -4– + 37 nt is the most important for expression [27], and furthermore, Goodman et al. and Bentele et al. demonstrated that rare codons are enriched at the N terminus of genes which reduces mRNA secondary structures in the translation initiation region [28, 29]. As the N-terminal signal sequences are generally located near the translation start site, it made sense to determine how the alteration of the codon sequence of the signal sequences affect the mRNA secondary structures in the translation start site. In this study, local strength of secondary structures (strength of the secondary structures are presented as Gibbs free energy) in the mRNAs were determined by using “mRNA profile v2” module of the Visual Gene Developer. The module calculates the strength of secondary structures for the first 30 bases and then the window proceeds base-by-base along the mRNA and calculates the strength of secondary structures for the next 30 bases. The analyzed region of the bicistronic mRNA covered the sequence starting from the *Lac* promoter and ending at last amino acid of the heavy chain (see Fig. 1). Like done above, Spearman correlation coefficient was calculated between predicted Gibbs free energy and the expression levels of immunoreactive Fab fragment at each base position to find out, which positions have a significant effect on the expression levels. After Bonferroni correction (alpha level = 0.0013), the sliding window analysis showed that the 30 nt region centered around 155–157 and 159–160 nt which located between the light chain SD and the start codon (hereafter referred as significant translation initiation region, significant TIR) of the N–0 library correlated significantly with the expression (Fig. 7). It was observed that reduced mRNA secondary structures of the significant TIR correlated with higher expression (positive Spearman rank correlation coefficient). One significant 30 nt region that was centered around 886 nt was found from the proximity of the heavy chain SD and unlike significant TIR of the light chain, the region centered around 886 nt correlated negatively (n = 30, r= − 573, *p* = 0.0009; Bonferroni corrected alpha level = 0.00128) with the expression, that is, lower Gibbs free energy (more stable secondary structures) resulted in higher expression. No correlations between positional Gibbs free energy and expression were found in H-0 and C-0 libraries.

Bioinformatics analysis revealed that there was a strong negative correlation (n = 30, r = − 0.845, *p *= 4.2 × 10^−9^) between the average Gibbs energy of mRNA secondary structure of the significant TIR (as analyzed with 30 nt window centered around nucleotide positions 155–157, 159–160) and codon optimality at position Leu-5. Especially the optimal codon CTG at Leu-5 position decreased the Gibbs free energy of mRNA secondary structures, which is logical since it is GC-rich. To see if the codon optimality at position Leu-5/mRNA secondary structure strength combination had additive independent effects on the expression levels and to exclude the effect of codon identity on the Gibbs free energy of the secondary structure, we pooled the clones in N–0 library according to the codon identity at position Leu-5 (CTA, CTC, CTG and CTT) like it was done above in the analysis of codon pools. Then we plotted the expression levels against the average Gibbs free energy of the TIR and analyzed the correlation with Spearman rank correlations coefficient in each pool. It was observed that especially in the CTA codon pool there was a strong and significant positive correlation (n = 6, r = 0.845, *p *= 0.034) between the average Gibbs free energy and the expression levels. Such correlation was not observed in other codon pools CTT (n = 7, r = 0.642, *p *= 0.120), CTC (n = 7, r = 0.600, *p *= 0.154) or CTG (n = 6, r = 0.525, *p *= 0.285). We further explored the relationship between the mRNA secondary structures in the significant TIR and the codon optimality at position Leu-5 by comparing previously introduced single codon variant Leu-5 CTA and new single codon variant Leu-5 TTA. Both variants have exactly the same Gibbs free energy profile of the mRNA according to the “mRNA profile v2” module of the Visual Gene Developer. However, CTA codon has the relative adaptiveness value (*w*_i_) of 0.0724, whereas TTA codon has the relative adaptiveness value (*w*_i_) of 0.2636. After 3 h of induction, it was observed that the single codon variant Leu-5 CTA exhibited 26% higher expression levels of immunoreactive Fab fragment than the single codon variant Leu-5 TTA. The difference between the variants was also statistically significant (five independent expressions) according to T-test (Equal variances assumed, Sig. 2-tailed *p* = 0.000004).

### Synonymous codon pairs are enriched in the hydrophobic region

**Fig. 8:**
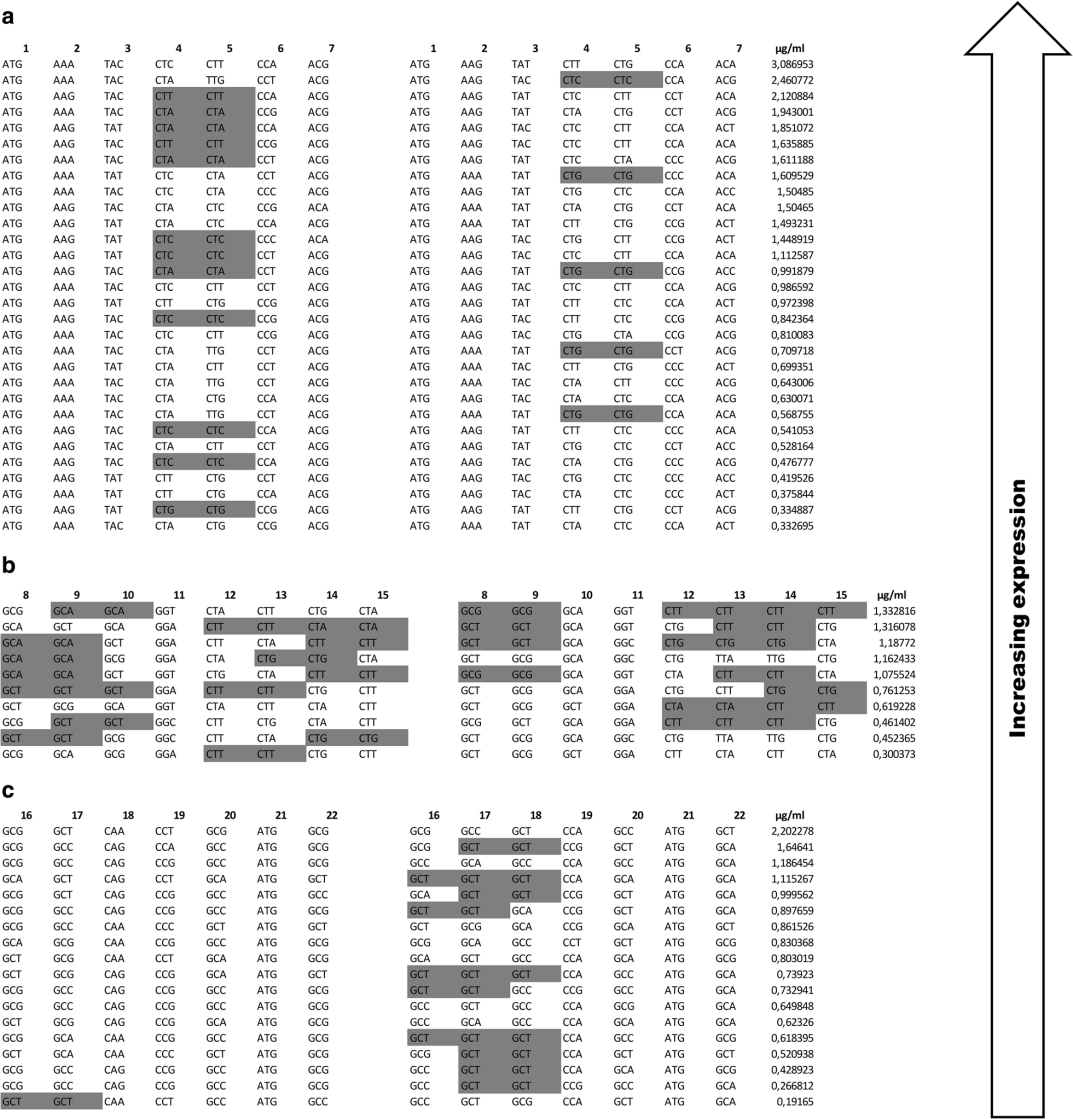
Relationship between synonymous codon pair bias and the expression levels of immunoreactive Fab fragment in **a** N–0 library, **b** H–0 library and **c** C–0 library. The clones of each library are sorted in descending order according to the expression levels of immunoreactive Fab fragment. Synonymous codon pairs are indicated by gray color. Light chain PelB sequences are on the left side and heavy chain PelB sequences on the right side

It was shown in yeast *Saccharomyces cerevisiae* that if the same amino acids reside next to each other, the latter one favors the same codon as the previous one [30]. Later this phenomenon, “synonymous codon pair bias”, was also detected in many bacterial species [31]. Since the PelB signal sequence includes leucine pair in the n-region, alanine and leucine runs in the hydrophobic region and alanine run in the c-region, we investigated if synonymous codon pair bias is observed in the clones of PelB signal sequence libraries. The synonymous codon pairs were detected in all libraries and in both PelBs, apart from the light chain PelB of the C–0 library (Fig. 8a–c). Biased use of synonymous codon pairs was observed in the case of alanine pair (A8–A9) of the heavy chain PelB of the H–0 library (Fig. 8b). The alanine pair [Ala-8]–[Ala-9] with synonymous codon pair GCT–GCT or GCG–GCG exhibited, on average, almost twofold higher expression levels of immunoreactive Fab fragment than the alanine pair without synonymous codon pair. The difference between the two groups, with and without the synonymous codon pair, was statistically significant according to the T-test (Equal variances assumed, Sig. 2-tailed *p* = 0.006). In general, the H–0 library was highly occupied with synonymous codon pairs compared to the other libraries. Of the H–0 library clones, 90% had synonymous codon pair in the light chain PelB and 70% had synonymous codon pair in the heavy chain PelB. Respective values for the N–0 library were 40% and 17% and for the C–0 library 6% and 56%.

### Up-scaled Fab fragment expression

To confirm that the results are applicable also in larger culture volume, the anti-digoxigenin Fab fragment was expressed in *E. coli* XL1-Blue in 500 ml culture volume with two different PelB codon sequences: the best library clone PelB codon sequence (N–0 2) (Additional file 1: Fig S1) and the parental PelB (the parent Fab0) codon sequence. Both expressions were implemented in triplicates. In order to determine the effect of codon usage on translocation efficiency of the Fab fragment, cells were fractionated to periplasmic fraction and cytoplasmic fraction. Like in 5 ml culture volume, the best library clone outperformed the parent Fab0 also in 500 ml culture volume. According to the immunoassay measurement of the periplasmic extract, the codon sequence of the best library clone PelB indeed enhanced the translocation of the immunoreactive Fab fragment to the periplasm. It was observed that in the case of the best library clone PelB, 78% of the total amount of the immunoreactive Fab fragment (1.97 µg in 1 ml volume) was found in the periplasm. The respective efficiency for the parental PelB was only 59% (0.56 µg in 1 ml volume). The difference in the amount of immunoreactive Fab fragment in the periplasm between the two sequences was also statistically significant (T-test, Equal variances assumed, Sig. 2-tailed *p* = 0.005).

### The best library clone light and heavy chain PelBs (N–0 2) together also improve the expression levels of other Fab fragments

From the versatility point-of-view, it was interesting to see if the best library clone PelB codon sequence increases the expression levels of other Fab fragments as well. To study this, we introduced the light chain and the heavy chain PelB codon sequences of the best library clone to an anti-microcystin Fab and to an anti-SORLA (sortilin related receptor) Fab and compared the expression levels to the same Fabs carrying the parental PelB signal sequences. The expressions and immunoassays (target molecule for anti-microcystin Fab = biotinylated microcystin MC-LR; target molecule for anti-SORLA Fab = sortilin related receptor fragment) were performed in the same manner as the secondary screenings described above. Indeed, the best library clone PelB codon sequences in the light and heavy chain increased the expression levels in both cases. The specific signal levels of the anti-microcystin Fab equipped with the best library clone PelBs increased by 3.9-fold when compared to the same Fab with parental PelBs. The expression levels of the anti-SORLA Fab equipped with parental PelBs was in fact so low that it couldn’t be reliably detected by the immunoassay. However, when the best library clone PelBs were introduced to this Fab, the expression levels increased to the measurable level (background vs. anti-SORLA Fab, T-test, Equal variances assumed, Sig. 2-tailed *p* = 0.001).

## Discussion

In the present study we introduced synonymous mutations into the PelB signal sequences of the light and heavy chain genes coding for a human anti-digoxigenin Fab molecule. The PelB signal sequence is composed of three structurally distinguishable regions that were diversified in parallel resulting in the signal sequence libraries N–0 (diversified n-region), H–0 (diversified hydrophobic region) and C–0 (diversified c-region). Furthermore, the equivalent regions (n, h or c) were simultaneously diversified in the light chain PelB and the heavy chain PelB. The screening of the three libraries with immunoassays showed that the N–0 library was the best source for obtaining clones with improved expression. In the N–0 library, 43% of the clones exhibited at least twofold increase in expression and 17% at least threefold increase in expression compared to the Fab0. The highest expressing variant exhibited over fivefold increase in expression. The respective percentages for twofold and threefold increases were 40% and 0% in the H–0 library and 22% and 6% in the C–0 library. However, 23%, 30% and 22% of the N–0, H–0 and C–0 library clones showed decreased expression in the secondary screening, which points towards the inherently high variation in the expression results in the primary screening campaign, which are caused by the challenges to normalize the cell number and growth phase of cells in 96-plate format.

A detailed analysis of positional effects of synonymous codon usage in PelB on Fab expression revealed that the identity of the fifth leucine codon in the light chain PelB affected the expression levels significantly. Especially CTG, the most optimal leucine codon, had deleterious effect on the expression, whereas the less optimal codons CTT and CTA had a beneficial effect on expression (Fig. 5). The finding was further confirmed with single codon variants of Leu-5 (Fig. 6) showing that the wild-type codon TTG does not yield the best result for Fab expression. Furthermore, the n-regions of the wild-type PelB and the parent PelB are identical (Fig. 2). The beneficial effect of non-optimal codons has been described earlier [11, 12, 32], but our results suggest that the position of non-optimal codon matters more than the mere number of non-optimal codons since the non-optimal codon triplet [Leu-4]-[Leu-5]-[Pro-6] did not increase the expression levels (Fig. 6). Moreover, we did not observe any significant correlation between lower CAI values and increased expression levels.

Previously, it has been shown that the folding energy of mRNA secondary structures in the translation initiation region can have a major impact on expression and that non-optimal/rare codons are enriched at the N-terminus of genes, which typically reduce mRNA secondary structures in the translation initiation region as AT-rich codons [27–29]. Our findings support these results as reduced folding energy of mRNA secondary structures at the translation initiation region of the light chain coincided with increased Fab expression (Fig. 7). However, we found that the N-terminal codon usage might also have an effect on expression, which is independent from the mRNA secondary structure. The single codon variant Leu-5 CTA and Leu-5 TTA had identical Gibbs free energy profiles indicating similar mRNA secondary structures, but the Fab expression of Leu-5 CTA variant was 26% higher than that of Leu-5 TTA at 3 h time point post induction. Zalucki et al. hypothesized that the combination of rapid translation initiation and non-optimal codons in the leader peptide could be related to higher efficiency in protein translocation or recycling of chaperones [33]. Our results suggest that reduced mRNA secondary structure at the translation initiation site and non-optimal codon usage at a particular position can have additive independent roles on protein translocation efficiency. Regarding the relationship between the mRNA secondary structure and the expression levels, it was interesting that the 30 nt region centered around position 886 nt, which was located just before the translation initiation region of the heavy chain, increased mRNA secondary structures correlated with increased expression levels.

In the H–0 library, only significant factor explaining the expression difference was the usage of synonymous codon pairs. In fact, the synonymous codon pairs were highly enriched in the H–0 library when compared to the other libraries (Fig. 8). This enrichment might be related to structural sensitivity of the regions with high hydrophobicity. Pechmann and Frydman showed that optimal codons, which are translated faster, are enriched in the hydrophobic regions [34]. In addition, Cannarrozzi et al. found that usage of synonymous codon pairs can increase translation speed up to 30% [30]. Therefore, it is plausible that enrichment of synonymous codon pairs is due to the structural sensitivity of the hydrophobic region. It is worth noticing that either hydrophobic region of the heavy chain of the parent PelB or the wild-type PelB don’t contain any synonymous codon pairs (Fig. 2). Moreover, we demonstrate that improved PelB codon sequence obtained from the library screening enhances the expression of the Fab fragment also in 500 ml culture scale, and in addition, in combination with different Fab genes.

## Conclusions

We established three synonymous signal sequence libraries which proved to be good sources for discovering Fab fragments with improved expression profile. When analyzing sequence parameters of clones obtained from the synonymous signal sequence libraries, we identified different factors that affect the expression of Fab fragment. Codon usage, especially avoidance of optimal CTG codon, at fifth leucine position of light chain PelB turned out to be highly important in terms of Fab fragment expression. Furthermore, we discovered that codon usage of fifth leucine position and the mRNA secondary structures of the translation initiation site exhibit independent additive effects on the expression of Fab fragment. In addition to aforementioned findings, we observed significant effects of mRNA secondary structures and synonymous codon pairs on the expression of Fab fragment.

## Supplementary information

**Additional file 1: Table S1.** Primers used in the diversification of the regions of the PelB signal sequence. **Fig. S1.** Codon sequences of the analyzed library clones. The light chain PelB codon sequences of the variants are shown in the left column and the heavy chain PelB codon sequences of the variants are shown in the right column.

## Data Availability

All data generated or analyzed during this study are included in the published article (and its additional file).
