# Supplementary material for: Improvement of Fab expression by screening combinatorial synonymous signal sequence libraries
Source: Microb Cell Fact. 2019 Sep 16;18:157. doi: 10.1186/s12934-019-1210-1 (PMC6745802; doi:10.1186/s12934-019-1210-1)
Supplement: Supplementary file 1 — Additional file 1: Table S1. Primers used in the diversification of the regions of the PelB signal sequence. Fig. S1. Codon sequences of the analyzed library clones. The light chain PelB codon sequences of the variants are shown in the left column and the heavy chain PelB codon sequences of the variants are shown in the right column. [file 12934_2019_1210_MOESM1_ESM.pdf]

Additional file

Improvement of Fab expression by screening combinatorial synonymous signal sequence libraries

Antti Kulmala[1], Tuomas Huovinen[1] and Urpo Lamminmäki[1,*]

[1] Department of Biochemistry/Biotechnology, University of Turku, Kiinamyllynkatu 10, 20520 Turku, Finland

*Corresponding author

To whom correspondence should be addressed:

Urpo Lamminmäki

E-mail: urpo.lamminmaki@utu.fi.

Tel: +358-29-450 4586

Table S1. Primers used in the diversification of the regions of the PelB signal sequence

Primer name	Primer sequence
**Light chain PelB ss n-region primer**	tagataacgagggcaaatcATGAARTAYCTNCTNCCNACNgcagccgctggattgttattactcg
**Heavy chain PelB ss n-region primer**	tctagattaattaaaggagaattgaatATGAARTAYCTNCTNCCNACNgctgcggcaggcctgttattg
**Light chain PelB ss hydrophobic region primer**	catgaaatacctattgcctacgGCDGCDGCDGGHCTDCTDCTDCTDgcggcccagccggccatggcg
**Heavy chain PelB ss hydrophobic region primer**	tatgaaatatcttctgccgactGCDGCDGCDGGHCTDCTDCTDCTDgcggccgctccagccatggctgagg
**Light chain PelB ss c-region primer**	gccgctggattgttattactcGCNGCNCARCCNGCNATGGCNgagatcgtgctgacccaatctc
**Heavy chain PelB ss c-region primer**	gcaggcctgttattgctgGCNGCDGCNCCNGCNATGGCNgaggtacagctgcttgaaagcg

Library	Clone	Light chain 1	2	3	4	5	6	7	8	Heavy chain 1	2	3	4	5	6	7	8
N-0 library	N-01	ATG	AAG	TAC	CTT	CTT	CCA	ACG		ATG	AAG	TAT	CTC	CTT	CCT	ACA	
	N-02	ATG	AAA	TAC	CTC	CTT	CCA	ACG		ATG	AAG	TAT	CTT	CTG	CCA	ACA	
	N-03	ATG	AAG	TAC	CTC	CTC	CCG	ACG		ATG	AAG	TAC	CTT	CTC	CCG	ACG	
	N-04	ATG	AAG	TAT	CTA	CTA	CCA	ACG		ATG	AAG	TAC	CTC	CTT	CCA	ACT	
	N-05	ATG	AAG	TAT	CTC	CTC	CCC	ACA		ATG	AAG	TAC	CTG	CTT	CCG	ACT	
	N-06	ATG	AAA	TAT	CTC	CTA	CCT	ACG		ATG	AAA	TAT	CTG	CTG	CCC	ACA	
	N-07	ATG	AAG	TAT	CTC	CTC	CCT	ACG		ATG	AAG	TAC	CTC	CTT	CCA	ACA	
	N-08	ATG	AAG	TAC	CTA	CTT	CCT	ACG		ATG	AAG	TAT	CTG	CTC	CCT	ACC	
	N-09	ATG	AAA	TAC	CTA	TTG	CCT	ACG		ATG	AAG	TAC	CTC	CTC	CCA	ACG	
	N-010	ATG	AAG	TAC	CTT	CTT	CCG	ACG		ATG	AAG	TAC	CTC	CTT	CCA	ACA	
	N-011	ATG	AAG	TAC	CTA	CTA	CCT	ACG		ATG	AAG	TAT	CTG	CTG	CCG	ACC	
	N-012	ATG	AAA	TAC	CTA	CTA	CCT	ACG		ATG	AAG	TAT	CTC	CTA	CCC	ACG	
	N-013	ATG	AAA	TAC	CTC	CTT	CCT	ACG		ATG	AAG	TAC	CTC	CTT	CCA	ACG	
	N-014	ATG	AAG	TAT	CTT	CTG	CCG	ACG		ATG	AAG	TAT	CTT	CTC	CCA	ACT	
	N-015	ATG	AAA	TAC	CTC	CTA	CCC	ACG		ATG	AAG	TAT	CTG	CTC	CCA	ACC	
	N-016	ATG	AAA	TAT	CTT	CTG	CCA	ACG		ATG	AAG	TAC	CTA	CTC	CCC	ACT	
	N-017	ATG	AAA	TAC	CTA	TTG	CCT	ACG		ATG	AAA	TAC	CTA	CTT	CCC	ACG	
	N-018	ATG	AAA	TAC	CTC	CTT	CCG	ACG		ATG	AAG	TAC	CTG	CTA	CCG	ACG	
	N-019	ATG	AAA	TAC	CTA	CTG	CCG	ACG		ATG	AAA	TAT	CTA	CTC	CCA	ACT	
	N-020	ATG	AAG	TAT	CTA	CTC	CCA	ACG		ATG	AAA	TAT	CTT	CTG	CCG	ACT	
	N-021	ATG	AAG	TAC	CTC	CTC	CCA	ACG		ATG	AAG	TAT	CTT	CTC	CCC	ACA	
	N-022	ATG	AAA	TAC	CTA	TTG	CCT	ACG		ATG	AAA	TAT	CTG	CTG	CCA	ACA	
	N-023	ATG	AAA	TAC	CTA	TTG	CCT	ACG		ATG	AAA	TAT	CTG	CTG	CCT	ACG	
	N-024	ATG	AAG	TAC	CTC	CTC	CCA	ACG		ATG	AAG	TAC	CTA	CTG	CCC	ACG	
	N-025	ATG	AAG	TAC	CTA	CTC	CCG	ACA		ATG	AAA	TAT	CTA	CTG	CCT	ACA	
	N-026	ATG	AAA	TAC	CTA	CTA	CCG	ACG		ATG	AAG	TAT	CTA	CTG	CCT	ACG	
	N-027	ATG	AAG	TAT	CTG	CTG	CCG	ACG		ATG	AAG	TAT	CTT	CTG	CCT	ACG	
	N-028	ATG	AAG	TAT	CTA	CTT	CCT	ACG		ATG	AAA	TAC	CTT	CTG	CCC	ACT	
	N-029	ATG	AAA	TAC	CTA	CTG	CCA	ACG		ATG	AAG	TAC	CTA	CTC	CCA	ACG	
	N-030	ATG	AAG	TAT	CTT	CTG	CCT	ACG		ATG	AAG	TAC	CTG	CTC	CCC	ACC	
H-0 library	H-01	GCG	GCA	GCA	GGT	CTA	CTT	CTG	CTA	GCG	GCG	GCA	GGT	CTT	CTT	CTT	CTT
	H-02	GCA	GCA	GCT	GGT	CTG	CTA	CTT	CTT	GCG	GCG	GCA	GGT	CTA	CTT	CTT	CTA
	H-03	GCA	GCT	GCA	GGA	CTT	CTT	CTA	CTA	GCT	GCT	GCA	GGT	CTG	CTT	CTT	CTG
	H-04	GCG	GCT	GCT	GGC	CTT	CTG	CTA	CTT	GCG	GCT	GCA	GGA	CTT	CTT	CTT	CTG
	H-05	GCT	GCT	GCT	GGA	CTT	CTT	CTG	CTT	GCT	GCG	GCA	GGA	CTG	CTT	CTG	CTG
	H-06	GCT	GCG	GCA	GGT	CTA	CTT	CTA	CTT	GCT	GCG	GCT	GGA	CTA	CTA	CTT	CTT
	H-07	GCT	GCT	GCG	GGC	CTT	CTA	CTG	CTG	GCT	GCG	GCA	GGC	CTG	TTA	TTG	CTG
	H-08	GCA	GCA	GCT	GGA	CTT	CTA	CTT	CTT	GCT	GCT	GCA	GGC	CTG	CTG	CTG	CTA
	H-09	GCG	GCA	GCG	GGA	CTT	CTT	CTG	CTT	GCT	GCG	GCT	GGA	CTT	CTA	CTT	CTA
	H-010	GCA	GCA	GCG	GGA	CTA	CTG	CTG	CTA	GCT	GCG	GCA	GGC	CTG	TTA	TTG	CTG
C-0 library	C-01	GCG	GCC	CAA	CCC	GCT	ATG	GCT		GCT	GCG	GCA	CCG	GCA	ATG	GCT	
	C-02	GCG	GCC	CAG	CCG	GCC	ATG	GCG		GCC	GCA	GCC	CCA	GCC	ATG	GCA	
	C-03	GCG	GCC	CAG	CCA	GCC	ATG	GCG		GCG	GCT	GCT	CCG	GCT	ATG	GCA	
	C-04	GCG	GCC	CAG	CCG	GCC	ATG	GCG		GCT	GCT	GCA	CCG	GCT	ATG	GCA	
	C-05	GCA	GCT	CAG	CCT	GCA	ATG	GCT		GCT	GCT	GCT	CCA	GCA	ATG	GCA	
	C-06	GCG	GCT	CAA	CCT	GCG	ATG	GCG		GCG	GCC	GCT	CCA	GCC	ATG	GCT	
	C-07	GCT	GCG	CAG	CCG	GCA	ATG	GCT		GCT	GCT	GCT	CCA	GCC	ATG	GCA	
	C-08	GCG	GCA	CAA	CCG	GCC	ATG	GCG		GCT	GCT	GCT	CCA	GCC	ATG	GCG	
	C-09	GCG	GCC	CAG	CCG	GCC	ATG	GCG		GCC	GCT	GCT	CCA	GCA	ATG	GCG	
	C-010	GCT	GCG	CAA	CCT	GCA	ATG	GCG		GCA	GCT	GCC	CCA	GCA	ATG	GCG	
	C-011	GCG	GCC	CAG	CCG	GCC	ATG	GCG		GCT	GCT	GCC	CCG	GCC	ATG	GCA	
	C-012	GCT	GCT	CAA	CCT	GCC	ATG	GCC		GCC	GCT	GCG	CCA	GCT	ATG	GCA	
	C-013	GCT	GCG	CAG	CCG	GCA	ATG	GCG		GCC	GCA	GCC	CCA	GCA	ATG	GCA	
	C-014	GCG	GCT	CAG	CCG	GCC	ATG	GCC		GCA	GCT	GCT	CCG	GCT	ATG	GCA	
	C-015	GCG	GCC	CAG	CCG	GCC	ATG	GCG		GCC	GCT	GCC	CCA	GCG	ATG	GCA	
	C-016	GCT	GCA	CAA	CCC	GCT	ATG	GCG		GCG	GCT	GCT	CCA	GCT	ATG	GCT	
	C-017	GCA	GCG	CAA	CCG	GCC	ATG	GCG		GCG	GCA	GCC	CCT	GCT	ATG	GCG	
	C-018	GCG	GCC	CAG	CCG	GCC	ATG	GCG		GCC	GCT	GCT	CCG	GCC	ATG	GCA	

Fig. S1. Codon sequences of the analyzed library clones. The light chain PelB codon sequences of the variants are shown in the left column and the heavy chain PelB codon sequences of the variants are shown in the right column.
